# Mutated KIT Tyrosine Kinase as a Novel Molecular Target in Acute Myeloid Leukemia

**DOI:** 10.3390/ijms23094694

**Published:** 2022-04-23

**Authors:** Seiichiro Katagiri, SungGi Chi, Yosuke Minami, Kentaro Fukushima, Hirohiko Shibayama, Naoko Hosono, Takahiro Yamauchi, Takanobu Morishita, Takeshi Kondo, Masamitsu Yanada, Kazuhito Yamamoto, Junya Kuroda, Kensuke Usuki, Daigo Akahane, Akihiko Gotoh

**Affiliations:** 1 Department of Hematology, Tokyo Medical University, 6-7-1 Nishi-Shinjuku, Shinjuku-ku, Tokyo 160-0023, Japan; katagiri@tokyo-med.ac.jp (S.K.); dakahane@tokyo-med.ac.jp (D.A.); akgotou@juntendo.ac.jp (A.G.); 2 Department of Hematology, National Cancer Center Hospital East, 6-5-1 Kashiwanoha, Kashiwa-shi, Chiba 277-8577, Japan; schi@east.ncc.go.jp; 3 Department of Hematology and Oncology, Osaka University Graduate School of Medicine, 2-2 Yamadaoka, Suita, Osaka 565-0871, Japan; kfukushi@bldon.med.osaka-u.ac.jp (K.F.); hiro@bldon.med.osaka-u.ac.jp (H.S.); 4 Department of Hematology and Oncology, University of Fukui Hospital, 23-3 Matsuoka Shimoaizuki, Eiheiji-cho, Yoshida-gun, Fukui 910-1193, Japan; hosono@u-fukui.ac.jp (N.H.); tyamauch@u-fukui.ac.jp (T.Y.); 5 Division of Hematology, Japanese Red Cross Nagoya First Hospital, 3-35 Michishita-cho, Nakamura-ku, Nagoya-shi, Aichi 453-8511, Japan; morishita-tak@nagoya-1st.jrc.or.jp; 6 Blood Disorders Center, Aiiku Hospital, 2-1 S4 W25 Chuo-ku, Sapporo, Hokkaido 064-0804, Japan; kondo@aiiku-hp.or.jp; 7 Department of Hematology and Cell Therapy, Aichi Cancer Center, 1-1 Kanokoden, Chikusa-ku, Nagoya, Aichi 464-8681, Japan; myanada@aichi-cc.jp (M.Y.); kyamamoto@aichi-cc.jp (K.Y.); 8 Division of Hematology and Oncology, Kyoto Prefectural University of Medicine, 465 Kajii-cho Kawaramachi-hirokoji, Kamigyo-ku, Kyoto 602-8566, Japan; junkuro@koto.kpu-m.ac.jp; 9 Department of Hematology, NTT Medical Center Tokyo, 5-9-22 Higashi-Gotanda, Shinagawa-ku, Tokyo 141-8625, Japan; kensuke.usuki@gmail.com

**Keywords:** acute myeloid leukemia, genome profiling, *KIT* mutation, *RUNX1*-*RUNX1T1*, HSP90 inhibitor

## Abstract

KIT is a type-III receptor tyrosine kinase that contributes to cell signaling in various cells. Since KIT is activated by overexpression or mutation and plays an important role in the development of some cancers, such as gastrointestinal stromal tumors and mast cell disease, molecular therapies targeting *KIT* mutations are being developed. In acute myeloid leukemia (AML), genome profiling via next-generation sequencing has shown that several genes that are mutated in patients with AML impact patients’ prognosis. Moreover, it was suggested that precision-medicine-based treatment using genomic data will improve treatment outcomes for AML patients. This paper presents (1) previous studies regarding the role of *KIT* mutations in AML, (2) the data in AML with *KIT* mutations from the HM-SCREEN-Japan-01 study, a genome profiling study for patients newly diagnosed with AML who are unsuitable for the standard first-line treatment (unfit) or have relapsed/refractory AML, and (3) new therapies targeting *KIT* mutations, such as tyrosine kinase inhibitors and heat shock protein 90 inhibitors. In this era when genome profiling via next-generation sequencing is becoming more common, *KIT* mutations are attractive novel molecular targets in AML.

## 1. Introduction

KIT is a type-III receptor tyrosine kinase that contributes to signal transduction in certain cells, such as hematopoietic stem cells, mast cells, and Cajal cells of the gastrointestinal tract [1]. *KIT* mutations have been reported in more than 90% of cases of mast cytosis [2,3], 80–85% of cases of gastrointestinal stromal tumor (GIST) [4], 10–20% of cases of melanoma [5,6], and cases of acute myeloid leukemia (AML), especially in core-binding factor (CBF) leukemia [7,8,9,10,11]. In AML, recent studies involving the genome profiling of AML via next-generation sequencing (NGS) showed that some mutated genes (e.g., *ASXL1*, *NPM1*, *FLT3*, *TP53*, *CEBPA*, and *RUNX1*) in patients with AML impacted the prognosis of these patients [12,13,14]. Moreover, recent clinical studies incorporating genomic data into treatment decisions, such as the BEAT AML trial [15], suggested that precision-medicine-based treatment using genomic data will improve treatment outcomes for AML. In this era when NGS genome profiling is becoming more common, *KIT* mutations are attracting attention as new molecular targets in AML.

## 2. Structure, Function, and Mutation of *KIT*

### 2.1. Structure and Function of KIT

**Figure 1:**
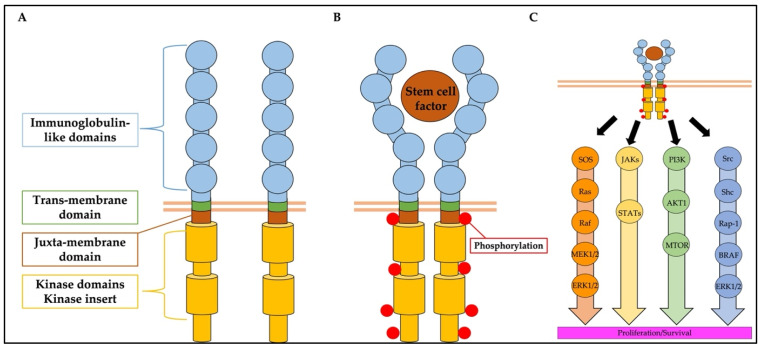
(**A**) Schematic representation of the structure of KIT. (**B**) The homodimeric state of KIT brought about by SCF binding and stabilized by interactions between immunoglobulin-like domains. (**C**) Signaling pathways involving KIT. The MAPK pathway, JAK/STAT pathway, P13K pathway, and Src family kinase pathway are shown as orange, yellow, green, and blue lines, respectively.

The *KIT* gene is located on chromosome segment 4q11 in humans and is composed of 21 exons [3,16]. The structure of KIT consists of five immunoglobulin-like (Ig-like) domains (D1-D2-D3-D4-D5), a trans-membrane domain (TMD), a juxta-membrane domain (JMD), two kinase domains (KD), and a kinase insert that lies between the KDs [17] (Figure 1A). KIT is expressed on the cell surface and functions as a receptor. The first three Ig-like domains (D1-D3) bind the stem cell factor (SCF), and the two KIT monomers are adjacent to each other. After that, the interaction between D4-D4 and D5-D5 occurs between adjacent KIT monomers, and a stable homodimer is formed. It generates trans-phosphorylation in the JMD region, kinase insert region, KD, and COOH-terminal tail (Figure 1B) [3,18,19]. The signals transmitted by KIT activation are primarily mediated through the phosphatidylinositol 3-kinase (P13K) pathway [20,21], Janus kinase (JAK)/signal transducers and activators of transcription (STAT) pathway [22,23,24], MAPK pathway [25,26,27], and the Src family kinase pathway [26,28] (Figure 1C). In the hematopoietic system, KIT is strongly expressed in hematopoietic stem cells and progenitor cells [29]. KIT plays an important role in the self-renewal potency of hematopoietic stem cells and differentiation into myeloid and lymphoid cells [30,31]. The expression of KIT is observed to decrease with the differentiation of hematopoietic cells [32]; however, it is highly expressed in mast cells [33].

### 2.2. Mutations of KIT in Cancer

Both the downregulation and upregulation of KIT signaling have been reported in human cancers. In many cancers, such as GIST, mast cytosis, and AML, the activation of KIT was detected through overexpression or mutation [34]. Moreover, the downregulation of KIT signaling was detected in melanoma [35]. *KIT* mutations often occur in the membrane proximal immunoglobulin-like domain (exon 8 and exon 9), the JMD (exon 11), and the tyrosine kinase domain (exon 17) [36]. Mutations in the JMD of KIT have been described in GIST [37] and extranodal NK/T cell lymphoma (ENKL) [38]. Mutations in the tyrosine kinase domain of KIT are detected frequently in systemic mastocytosis (SM) [39,40], ENKL [38], and seminomas [41] (Table 1).

## 3. Prognosis of AML with *KIT* Mutations Treated with Conventional Chemotherapy

*KIT* mutations are detected in approximately 4–6% of adult patients with de novo AML [13,42] and 20–40% of adult patients with de novo CBF-AML [7,8,9,10,11]. Fan et al. reported that 256 patients (23%) had *KIT* mutations in 1123 children with CBF-AML [43]. Three mutational hot-spots (exon 8, exon 10–11, and exon 17) have been identified in the *KIT* gene [44,45,46,47] (Table 2). Of these, exon 17 has been recognized as the site of *KIT* mutations most strongly associated with poor prognosis in adult patients with de novo AML harboring *RUNX1-RUNX1T1* [7,11,48,49]. Ishikawa et al. showed that *KIT* exon-17 mutations were associated with poor prognoses in patients with de novo AML with *RUNX1-RUNX1T1* being treated with an HDAC regimen [49].

Several reasons have been proposed for the poor prognosis in AML with *RUNX1*-*RUNX1T1* harboring a *KIT* mutation. For instance, it has been reported that activated KIT cooperates with a C-terminal truncated variant of RUNX1T1 to expand the pool of human CD34+ hematopoietic progenitors and augment the DNA repair machinery, resulting in increased chemo-resistance [50]. Using an in vitro model, Omori et al. compared the cell-proliferative and anti-apoptotic activity of *KIT*-D816V and *KIT*-N822K, both of which have been shown to undergo autophosphorylation in the absence of growth factors. Cells harboring *KIT*-D816V exhibited the activation of the SRC kinase and JAK/STAT pathways and demonstrated greater cell-proliferative and anti-apoptotic ability than cells harboring *KIT*-N822K [51]. In another study, Tarlock et al. used a cell line harboring a *KIT* mutation in an in vitro functional analysis, confirming the results of a clinical study of pediatric CBF-AML [52]. Those authors showed that *KIT* exon-17 mutations resulted in aberrant KIT phosphorylation and were associated with worse clinical outcomes. They further reported that *KIT* exon-8 mutations have no functional or prognostic impact.

## 4. *KIT* Mutation in Unfit and Relapsed/Refractory AML: Results from the HM-SCREEN-Japan-01 Study

Hematologic Malignancy (HM)-SCREEN-Japan-01 (UMIN000035233) is a genome profiling study of patients newly diagnosed with adult AML who are unsuitable for the standard first-line treatment (unfit) or have relapsed/refractory (R/R) AML [53,54,55] (methods are described in Supplementary Materials). The objective of the present study was to evaluate the frequency and characteristics of cancer-related genomic alterations in patients with AML using a comprehensive genome profiling assay (FoundationOne^®^Heme (F1H)) and to determine the quality of specimens used in gene analysis. One hundred and eighty-two patients were recruited, and an F1H report was successfully obtained for one hundred and seventy-seven patients [53,55]. We show the subgroup analysis of the HM-SCREEN-Japan-01 dataset focusing on *KIT* mutations below.

### 4.1. Frequency of KIT Mutation in Unfit and R/R AML

**Figure 2:**
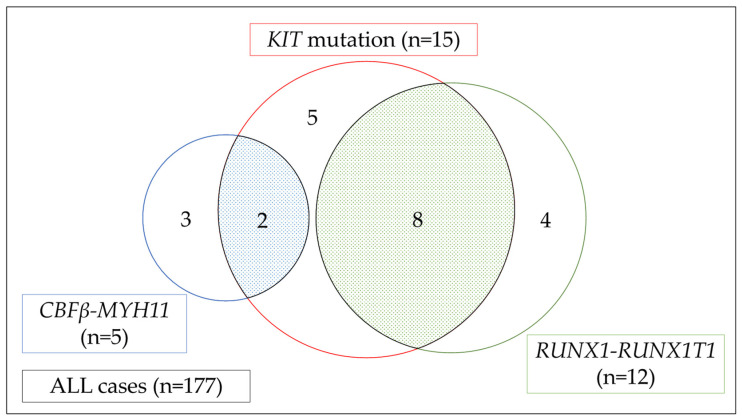
Venn diagram for frequency of *KIT* mutations and CBF leukemia in HM-SCREEN-Japan-01 [53,54,55]. Red, blue, and green circles indicate the number of patients with AML who harbored *KIT* mutation, *CBFβ*-*MYH11*, and *RUNX1*-*RUNX1T1*, respectively. Fifteen had the *KIT* mutation, eight of whom had *RUNX1*-*RUNX1T1* and two had *CBFβ*-*MYH11*.

Of the 177 patients who participated in the study, we identified 15 patients (8.5%) with a *KIT* mutation. Of the 15 AML patients with a *KIT* mutation, 6 were registered as unfit AML and 9 as R/R AML. In addition, a total of 17 patients with CBF leukemia (12 AML with *RUNX1*-*RUNX1T1* gene fusion and 5 AML with *CBFβ*-*MYH11* gene fusion) were confirmed via NGS analysis. Eight of the patients had both a *KIT* mutation and *RUNX1-RUNX1T1*; these individuals represented 53% of AML with *KIT* mutation cases and 67% of AML with *RUNX1*-*RUNX1T1* cases. Two patients had both a *KIT* mutation and *CBFβ*-*MYH11*; these individuals represented 13% of AML with *KIT* mutation cases and 40% of AML with *CBFβ*-*MYH11* cases. Five patients with non-CBF leukemia had a *KIT* mutation (Figure 2).

Our study showed a high frequency of *KIT* mutations in R/R or unfit CBF-AML patients compared with the previous studies targeting new-onset CBF-AML (Table 3). Patients’ characteristics and clinical outcomes are described in the Supplementary Materials.

### 4.2. Landscape of Gene Mutations in the KIT Mutation Cohort

**Figure 3:**
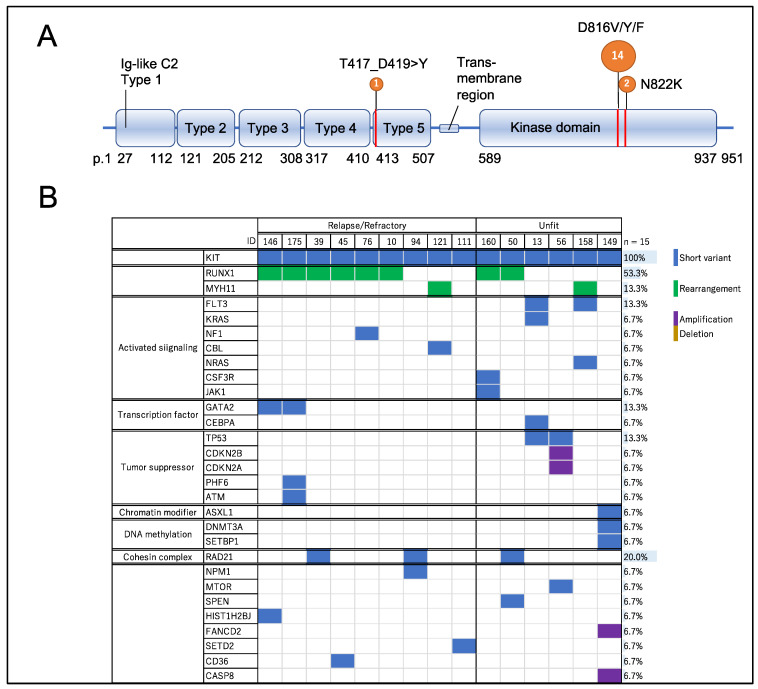
(**A**) Details of KIT mutation locations detected in HM-SCREEN-Japan-01 [53,54,55]. A total of 17 KIT mutations were detected in 15 cases. (**B**) Mutational data of the 15 patients with KIT mutations.

Fourteen of fifteen patients had a mutation in the region encoding the tyrosine kinase domain, resulting in predicted amino acid substitutions such as D816V, D816F, and N822K (Figure 3A). Two individuals (Patients 39 and 160) had two mutations in the region encoding the kinase domain (Table 4). The median variant allele frequency was 0.25. Chromosomal karyotypes were reported by each investigator. Eight cases were t(8;21)(q22;q22.1), and two were inv(16) or t(16;16). These rearrangements were confirmed via the detection of RUNX1-RUNX1T1 or CBFβ-MYH11, respectively, on NGS analysis. Of the five patients with non-CBF leukemia, two had a complex karyotype, and one had a 3q abnormality (Table 4). The mutation profiles for each case with a KIT mutation are shown in Figure 3B. The proportions of AML with RUNX1-RUNX1T1 among unfit and R/R patients harboring a KIT mutation were 19% (two of six) and 66% (six of nine), respectively. Other than KIT, the mutated gene detected most frequently was RAD21 (3/15, 20%). FLT3, TP53, and GATA2 mutations were found in two cases each (12%). The FLT3 mutations detected in patients 13 and 158 were FLT3-N676K and FLT3-D835H, respectively. The two cases with complex chromosomal abnormalities (patients 13 and 56) both harbored TP53 mutations. In R/R patients, mutations in tyrosine kinase-encoding genes other than KIT (e.g., FLT3 and JAK2) were not detected (Table 4).

### 4.3. Clinical Impact of KIT Mutation in Unfit and R/R AML

Our data also showed that AML with RUNX1-RUNX1T1 accounted for a very high proportion of unfit and R/R AML patients who had a KIT mutation. Notably, the proportion of AML with RUNX1-RUNX1T1 in R/R patients was 66%. Of the nine R/R patients, none harbored mutations in tyrosine kinase-encoding genes other than KIT, and the number of other gene mutations was similar in patients with and without RUNX1-RUNX1T1 (Figure 3B). Moreover, all of the KIT mutations detected in these nine R/R patients were located in exon 17 (typically encoding D816V/Y/F substitutions in the KIT protein). These data suggested that KIT mutations, especially those in exon 17, are related to a poor prognosis in AML with RUNX1-RUNX1T1, consistent with previous reports on the genetic profiling of R/R AML in patients with de novo AML [7,11,48,49,52].

Based on our analysis of all cases in HM-SCREEN-Japan-01, KIT mutations represented the predictor with the worst outcomes of all assessed gene mutations [53,55]. Most of the surviving patients had received allo-HSCT, regardless of whether they had been diagnosed with CBF or non-CBF leukemia in R/R cases (Supplementary Materials). However, CBF-AML is not currently indicated for transplantation after a first remission [14]. Indeed, there are unmet needs for these R/R patients, such as bridge therapy to transplantation.

In non-CBF leukemia with KIT mutation, three of five patients (Nos. 13, 56, and 111) had high-risk chromosomal abnormalities such as complex events or 3q abnormalities. An additional patient (No. 149) harbored t(3;3)(p25;q13), the source of which was unclear but might be related to the 3q abnormality. Although this subset of patients is small in number, these observations raise the interesting question of whether KIT mutations are associated with an elevated risk of chromosomal abnormality in non-CBF leukemia.

The results obtained from our study are limited by the small number of the KIT-mutated cases, and the results should be confirmed by increasing the total number of patients with AML including KIT-mutated AML. However, previous studies and our results suggested that the treatment strategies with conventional chemotherapy may not be able to overcome KIT-mutation-positive AML. Thus, new treatment agents targeting cancers with *KIT* mutations are needed.

## 5. Possible Role for Kinase Inhibitors in the Treatment of AML with *KIT* Mutation

Few specific inhibitors of KIT have been reported; however, several agents designed to target other RTKs such as FLT and ABL are expected to have utility for *KIT* mutations [56,57] (Table 5). Several drugs have been used in clinical trials in AML with KIT expression or *KIT* mutation.

Imatinib (IM), which inhibits ABL, KIT, and PDGFR, has been used in chronic myeloid leukemia, Philadelphia chromosome-positive acute lymphoblastic leukemia, and chronic eosinophilic leukemia with *PDGFRα* rearrangement. In a phase I study, a combination of cytarabine, daunorubicin, and IM was investigated in relapsed AML patients with KIT expression [58]. The complete remission (CR)/CR with incomplete platelet recovery (CRp) rate was 57%. In addition, the phase I/II study evaluated IM combined with mitoxantrone, etoposide, and cytarabine therapy for patients with R/R KIT-positive AML [59]. The combination was well tolerated up to 400 mg/day IM. Of the 21 patients treated at this dose, 13 (62%) achieved CR. Low-dose cytarabine (LDAC) and IM were well tolerated in incompatible or R/R AML patients with KIT expression [60]. However, the combination of LDAC and IM was not shown to be effective compared to LDAC monotherapy. Recently, it was reported that IM as maintenance therapy after the completion of post-remission therapy may improve the outcome of newly diagnosed AML patients [61].

Dasatinib is a medication that is expected to target cancers harboring *KIT* mutations [52,62,63]. Tarlock et al. showed that cells with *KIT* exon-17 mutations exhibited in vitro sensitivity to dasatinib [52]. In other work, Malani et al. obtained drug response profiles for established AML cell lines and ex vivo samples from patients with AML by subjecting the cells to high-throughput drug sensitivity and resistance testing with 290 approved and investigational oncology compounds [63]. They suggested that the gene-expression-based upregulation of the KIT pathway may serve as a biomarker of dasatinib efficacy in AML. Indeed, several clinical studies have examined the use of dasatinib for the treatment of CBF leukemia with a *KIT* mutation. For instance, in single-arm studies by the Cancer and Leukemia Group B (CALGB), patients with CBF leukemia received combination treatment with dasatinib and chemotherapy including HDAC [64]. The results of that study showed that patients harboring tumors with a *KIT* mutation had disease-free survival and overall survival comparable to those observed for patients harboring tumors with wild-type *KIT* [64]. Separately, in the phase Ib/IIa study of the German–Austrian AML Study Group (AMLSG), dasatinib was added to intensive induction/consolidation chemotherapy and administered as a maintenance treatment for CBF leukemia [65]. The exploratory analysis of the *KIT* mutation in that trial showed that five of nine patients who exhibited *KIT* mutation in paired samples from the time of diagnosis and relapse had lost the variant at relapse, suggesting the possibility that dasatinib inhibited clones with *KIT* mutations.

Midostaurin is a first-generation FLT3 inhibitor that inhibits *FLT3*-ITD and TKD mutations [66]. It was reported that the *KIT*-D816V receptor expressed in Ba/F3 cells was sensitive to midostaurin [48]. A therapeutic effect of midostaurin is expected in *KIT* D816V mutation-positive mastocytosis [67,68]. A phase II study (MIDOKIT study: NCT01830361) has been conducted to investigate the additional effect of midostaurin on the treatment of t(8; 21) AML with *KIT* or *FLT3*-ITD mutations, and its results are awaited.

## 6. HSP90 Inhibitors for the Treatment of AML with *KIT* Mutation

**Figure 4:**
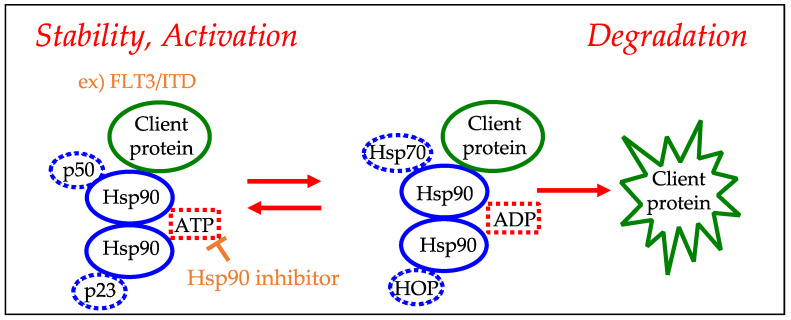
HSP90 inhibitor treatment for leukemia (such as FLT3). By binding of the HsP90 inhibitor to the ATP/ADP pocket of Hsp90, the equilibrium state of Hsp90 becomes ADP dominant. This inhibits the function of chaperone complexes containing client proteins and promotes the degradation of client proteins.

Heat shock protein 90 (HSP90) is a molecular chaperone that plays an important role in mediating the correct folding and functionality of its client proteins in cells [69,70]. HSP90 is involved in the stabilization of the cancer-related proteins necessary for tumor development, including receptor tyrosine kinases, signal transducers, cell-cycle regulators, and transcription factors [71,72]. Therefore, HSP90 inhibitors have been developed and are undergoing clinical trials in various cancers. One mechanism of HSP90 inhibitors is blocking the binding of ATP, which induces the degradation of target proteins [71,73,74] (Figure 4). In AML, it has been reported that HSP90 inhibitors may suppress mutated FLT3, as well as the JAK-STAT and P13K pathways [75,76,77]. Yu et al. reported that the inhibition of Hsp90 by 17-allylamino-17-demethoxygeldanamycin disrupted downstream signaling pathways of mutant KIT in a *RUNX1-RUNX1T1* with a *KIT*-mutant cell line [78]. Tsujimura et al. examined the potency of the novel KIT inhibitor KI-328 against different types of mutant KIT kinases in AML. They reported that KI-328 showed little potency against D816V-KIT; however, they demonstrated that HSP90 inhibitors suppress the growth of D816V-KIT-expressing cells [79]. Although these reports suggested the effect of HSP90 inhibitors on AML, the clinical use of HSP90 inhibitors has been delayed, partly due to their association with adverse events such as hepatotoxicity and visual abnormalities [72,74].

Pimitespib (TAS-116), a highly selective inhibitor of HSP90 α and β, is a new agent that is attracting attention for the treatment of malignancies with *KIT* mutations. HSP90 regulates the conformation, function, and activation of several HSP90 client proteins, including KIT [80,81]. In a mouse model, pimitespib showed anti-tumor activities while minimizing the adverse effects (e.g., visual disturbances) observed with other HSP90 inhibitors [72]. Pimitespib prolonged progression-free survival in a phase III trial comparing the efficacy and safety of pimitespib to a placebo in patients with previously treated GIST [82]. Recently, it was reported that pimitespib exhibits anti-adult T-cell leukemia/lymphoma (ATL) effects in ex vivo and in vivo preclinical models [74]. In this study, pimitespib suppressed the growth of ATL-related cell lines and primary ATL cells ex vivo and tumors in ATL cell-xenografted mice.

## 7. Conclusions

Here, we discussed the potential of *KIT* mutations as molecular targets for treating AML. KIT is a type-III receptor tyrosine kinase that contributes to signal transduction in many pathways, including the P13K, JAK/STAT, MAPK, and Src pathways, in various cells. The *KIT* mutation plays a central role in various malignant tumors such as GIST and SM, and it is attracting attention as an important molecular target. Treatment with tyrosine kinase inhibitors and HSP90 inhibitors is evolving for these diseases. In AML, it has been noted that the *KIT* mutation is associated with a poor prognosis in primary CBF leukemia. The HM-SCREEN01 study also showed that AML with *RUNX1*-*RUNX1T1* accounted for a very high proportion of patients with R/R AML with *KIT* mutations, but this point needs to be confirmed in the future by increasing the study population. Furthermore, with the development of NGS in recent years, the pathological and clinical roles of *KIT* mutations in AML other than CBF leukemia have also attracted attention. Current treatment strategies may not be able to overcome *KIT*-mutation-positive AML, and the availability of new precision medicine strategies targeting *KIT* mutations is eagerly awaited in clinical practice.

## Figures and Tables

**Table 1:** Summary of KIT mutations in cancers.

Site	Exon	Disease	Description
Immunoglobulin-like domain	8	AML	T417, Y418, D419
	9	GIST	A502
		Mastocytosis	K5091
Trans-membrane domain	10	AML	V530I
		Mastocytosis	F522C, A533D
Juxta-membrane domain	11	AML	V560, V559, ITD
		GIST	CD117, V559A, V559D, W557R, V560G
		Melanoma	L576P
		Mastocytosis	V560G
	13	AML	K642E
		Melanoma	K642E
	14	GIST	K704, N705
Kinase insert	15	GIST	S715
Kinase domain	16	AML	1748T, L773S
	17	AML	D816V, D816Y, D816F, D816H, N822, V8251
		Germ cell tumor	D816H, D816V
		Mastocytosis	D816V, D816Y, D816H, D820G
		ENKL	V825A, D816N

Abbreviations: AML: acute myeloid leukemia, GIST: gastrointestinal stromal tumor, ENKL: extranodal NK/T cell lymphoma.

**Table 2:** Summary of KIT mutations in AML.

Exon	Description	Functional Impact
8	T417, Y418, D419	Hyper-reactivity to stem cell factor
10–11	V530, V540, W557, V559, L576, ITD	Spontaneous dimer formation
17	D816, D820, N822, Y823, V825	Auto activation

Abbreviations: AML: acute myeloid leukemia.

**Table 3:** Frequency of KIT mutations in CBF-AML.

Author, Year	Disease Status	Frequency of *KIT* Mutations		
		CBF Leukemia	*RUNX1*-*RUNX1T1*	*CBFβ*-*MYH11*
Qin 2014	Newly diagnosed	37% (128/351)	39% (99/253)	30% (29/98)
Allen 2013	Newly diagnosed	28% (100/354)	23% (46/199)	35% (54/155)
Kim 2013	Newly diagnosed	26% (32/121)	27% (22/82)	35% (54/155)
Ishikawa 2019	Newly diagnosed	34% (63/199)	32% (42/132)	31% (21/67)
HM-SCREEN01	R/R or Unfit	59% (10/17)	67% (8/12)	40% (2/5)

Abbreviations: CBF: core-binding factor, R/R: relapse/refractory.

**Table 4:** Summary of KIT mutations and chromosomal karyotypes.

		*KIT* Mutation				
ID	Category	Description	SNV	VAF	Other Mutations	Chromosomal Karyotype
13	Unfit	D816V	2447A > T	0.372	FLT3, KRAS, CEBPA, TP53	Complex karyotype
50	Unfit	D816V	2447A > T	0.123	RAD21, SPEN	t(8;21)(q22;q22.1)
56	Unfit	D816F	2446_2447GA > TT	0.37	TP53, CDKN2A, CDKN2B	Complex karyotype
149	Unfit	D816V	2447A > T	0.344	ASXL1, DNMT3A, SETBP1, FANCD2, CASP8	46, XY, t(3;3)(p25:q13)
158	Unfit	T417_D419 > Y	1249_1255ACTTACG > T	0.078	FLT3, NRAS	inv(16)/t(16;16)
160	Unfit	D816V	2447A > T	0.146	CSF3R, JAK1	t(8;21)(q22;q22.1)
		N822K	2466T > G	0.018		
10	R/R	D816V	2447A > T	0.234	None	t(8;21)(q22;q22.1)
39	R/R	D816V	2447A > T	0.252	RAD21	t(8;21)(q22;q22.1)
		D816Y	2446G > T	0.056		
45	R/R	D816Y	2446G > T	0.923	CD36	t(8;21)(q22;q22.1)
76	R/R	D816V	2447A > T	0.932	NF1	t(8;21)(q22;q22.1)
94	R/R	D816V	2447A > T	0.459	RAD21, NPM1	Normal
111	R/R	D816V	2447A > T	0.338	SETD2	3q Abnormality
121	R/R	D816Y	2446G > T	0.021	CBL	inv(16)/t(16;16)
146	R/R	D816V	2447A > T	0.082	GATA2, HIST1H2BJ	t(8;21)(q22;q22.1)
175	R/R	N822K	2466T > G	0.461	GATA2, PHF6, ATM	t(8;21)(q22;q22.1)

Abbreviations: R/R: relapse/refractory, SNV: single-nucleotide variant, VAF: variant allele frequency.

**Table 5:** Summary of FDA-approved KIT-targeted therapies.

Drug	Primary Targets	FDA-Approved Disease
Imatinib	BCR-ABL1	CML, Ph+ALL, HES, GIST, SM, DFSP
Dasatinib	BCR-ABL1	CML, PhALL
Sunitinib	VEGFR and FLT3	GIST, RCC, Pancreatic Cancer
Regorafenib	VEGFR	GIST, HCC, Colorectal Cancer
Midostaurin	FLT3	AML (FLT3 mutation), SM
Ripretinib	KIT	GIST
Avapritinib	KIT/PDGFRA	GIST, SM

Abbreviations: FDA: US Food and Drug Administration, CML: chronic myeloid leukemia, PhALL: Philadelphia-positive acute lymphoblastic leukemia, HES: chronic eosinophilic leukemia with PDGFRα rearrangement, GIST: gastrointestinal stromal tumor, SM: systemic mastocytosis, DFSP: dermatofibrosarcoma protuberans, RCC: renal cell carcinoma, HCC: hepatocellular carcinoma.

## Data Availability

Data sharing not applicable.

## Supplementary Materials

The following supporting information can be downloaded at: https://www.mdpi.com/article/10.3390/ijms23094694/s1.
