# Supplementary material for: Mutated KIT Tyrosine Kinase as a Novel Molecular Target in Acute Myeloid Leukemia
Source: Int J Mol Sci. 2022 Apr 23;23(9):4694. doi: 10.3390/ijms23094694 (PMC9103326; doi:10.3390/ijms23094694)
Supplement: Supplementary file 1 [file ijms-23-04694-s001.zip › ijms-1659555-supplementary.pdf]

Supplemental materials.

Study design of HM-SCREEN-Japan 01

Hematologic Malignancy (HM)-SCREEN-Japan-01 is a multicenter genomic profiling study in which NGS by FoundationOne®Heme (F1H) was performed for patients with AML. The inclusion criteria were patients with R/R AML or with previously untreated AML who are ineligible for standard therapy (ND unfit). Available specimens consisted of paraffin-embedded bone marrow clots. Submission of archival specimens was allowed. After submission, annotated genomic reports were returned to the participants. Patients with *FLT3* mutations were allowed to submit specimen multiple times at different opportunities. Clinical information such as age, sex, treatment modality, response, stem cell transplantation status, and survival information was gathered. A total of seventeen faculties in Japan participated in this study; the National Cancer Center East serving as the representative site. The protocol was approved by each facility's institutional review board, and the study was conducted in accordance with the Declaration of Helsinki and subsequent amendments. All patients provided forms documenting their informed consent.

Comprehensive genome profiling assay: FoundationOne®Heme

F1H was developed by Foundation Medicine, Inc. (FMI), and provides a comprehensive genomic profile that applies NGS with a hybrid capture-based target enrichment approach, to identify somatic genomic alterations in genes known to be unambiguous drivers of hematologic malignancies (leukemias, lymphomas, and myelomas) and of sarcomas; the method uses formalin-fixed, paraffin-embedded specimens. Each profile simultaneously sequences the complete coding regions of 406 genes as well as the selected introns of 31 genes involved in chromosomal rearrangements. F1H also assesses the RNA sequences of transcripts from 265 commonly rearranged genes to better identify gene fusions (including de novo and rare gene fusions). In addition to detecting rearrangements, F1H detects all classes of genomic alterations, including base substitutions, insertion and deletions, and copy number alterations using a small, routine clinical sample. All F1H samples are profiled simultaneously for tumor mutation burden (TMB) status. TMB is determined by measuring the number of somatic mutations occurring in the sequenced genes on the F1H profile and extrapolating to the genome as a whole.

Statistical Analyses

Clinical information was summarized as descriptive statistics. Overall survival was displayed by the Kaplan-Meier method. The prognostic effect of basic characteristics (e.g., age, sex, and transplant status) and gene mutational status were calculated statistically by a Cox-regression model and summarized as the hazard ratio of death. The likelihood of co-occurrence in any pair of genes was expressed as the odds ratio of the observed number of duplications to the expected number of duplications. Given that the probability density of the odds ratio follows a Poisson distribution, the ratio was regarded as statistically significant if the upper-integrated probability was below 5%. The analyses were performed using R.

Patient characteristics of *KIT* mutation cohort in HM-SCREEN-Japan-01

Of 15 AML patients with a *KIT* mutation, 6 were registered as Unfit AML and 9 as R/R AML. Patients registered as Unfit were significantly older than patients registered as R/R (p=0.0024, Mann Whitney test). Eight patients with AML harbored *RUNX1-RUNTX1T1*. One case was

registered as AML with t(8;21)(q22;q22.1), but *RUNX1-RUNX1T1* was not detected by NGS analysis in this individual. No patients received an allogeneic hematopoietic stem cell transplant (allo-HSCT) before enrollment in the study. After study entry, some of patients with R/R AML received highly intensive salvage therapies such as mitoxantrone, etoposide, and cytarabine (MEC), high-dose cytarabine (HDAC), and idarubicine plus cytarabine (IDA/Ara-C); however, none of patients classified as Unfit received these intensive therapies (supplemental Table 1).

Supplemental Table S1. Patient characteristics of *KIT* mutation cohort

	Total (n=15)	Unfit (n=6)	R/R (n=9)
**Age (range)**	48 (22-91)	70 (60-91)	37 (22-72)
**PS (range)**	0 (0-3)	1 (0-3)	0 (0-1)
**Sex**			
Male	14 (93%)	6	8
Female	1 (7%)	0	1
**Initial diagnosis**			
AML with t(8;21)(q22;q22.1); *RUNX1-RUNX1T1*	9 (60%)	2	7
AML with inv(16)(p13.1q22) or t(16;16)(p13.1;q22); *CBFB-MYH11*	2 (13%)	1	1
AML with maturation	1 (7%)	0	1
Acute myelomonocytic leukemia	1 (7%)	1	0
AML with myelodysplasia-related changes	2 (13%)	2	0
**Previous allo-HSCT**			
No	15 (100%)	6	9
**Treatment after entry**			
MEC	1 (7%)	0	1
HDAC + MIT	1 (7%)	0	1
IDA + Ara-C	1 (7%)	0	1
Low dose IDA/DNR + Ara-C	5 (35%)	1	4
CA	2 (13%)	2	0
LDAC	1 (7%)	0	1
Azacitidine	3 (20%)	2	1
None	1 (7%)	1	0

Abbreviations: R/R: relapse/refractory, PS: performance status, AML: acute myeloid leukemia, allo-HSCT: allogeneic hematopoietic stem cell transplant, MEC: mitoxantrone, etoposide and cytarabine, HDAC: high dose cytarabine, MIT: mitoxantrone, Ara-C: cytarabine, IDA: idarubicine, DNR: daunorubicine, CA: cytarabine, aclarubicin, LDAC: low dose cytarabine

Clinical outcome in HM-SCREEN-Japan-01

The median overall survival (OS) of the 15 cases with *KIT* mutations was 854 days from the time of diagnosis (range, 50-2228 days) (Supplemental Figure 1A). There was no significant difference in OS between patients with CBF leukemia and other types (Supplemental Figure 1B). A Swimmer plot of the clinical course is shown in Supplemental Figure 2. All of the four patients enrolled as Unfit (Patients 13, 50, 158, and 160) died within 9 months of diagnosis and had shorter survival times than R/R patients who died (Patients 10, 39, 76, and 111). Three individuals enrolled as Unfit (Patients 13, 158, and 160) showed relapse or resistance to treatment. Death was confirmed in 7 of 12 cases who showed relapse or resistance to treatment,

with a median duration of 1.7 months from the last relapse to death. Eight patients received allo-HSCT. The median period from diagnosis to allo-HSCT was 16.4 months (range 2.9-25.8 months) and median survival period after allo-HSCT was 21.7 months (range 1.6-47.3 months). Two cases (Patients 10 and 111) relapsed within 12 months after allo-HSCT and died.

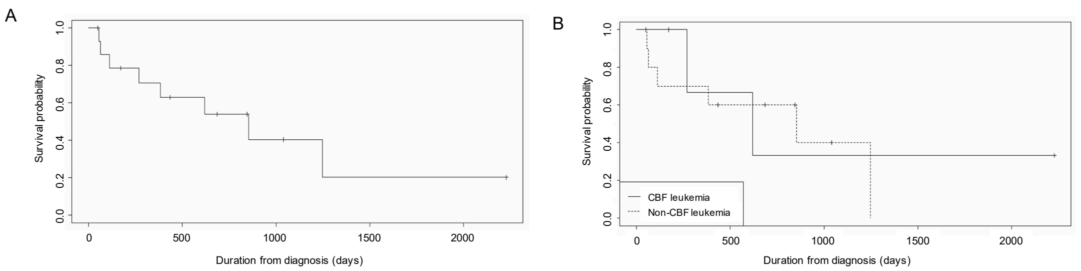

Supplemental Figure S1. (A) Overall survival of 15 patients with a *KIT* mutation. (B) Kaplan-Meier estimates of overall survival based on classification as CBF leukemia and non-CBF leukemia

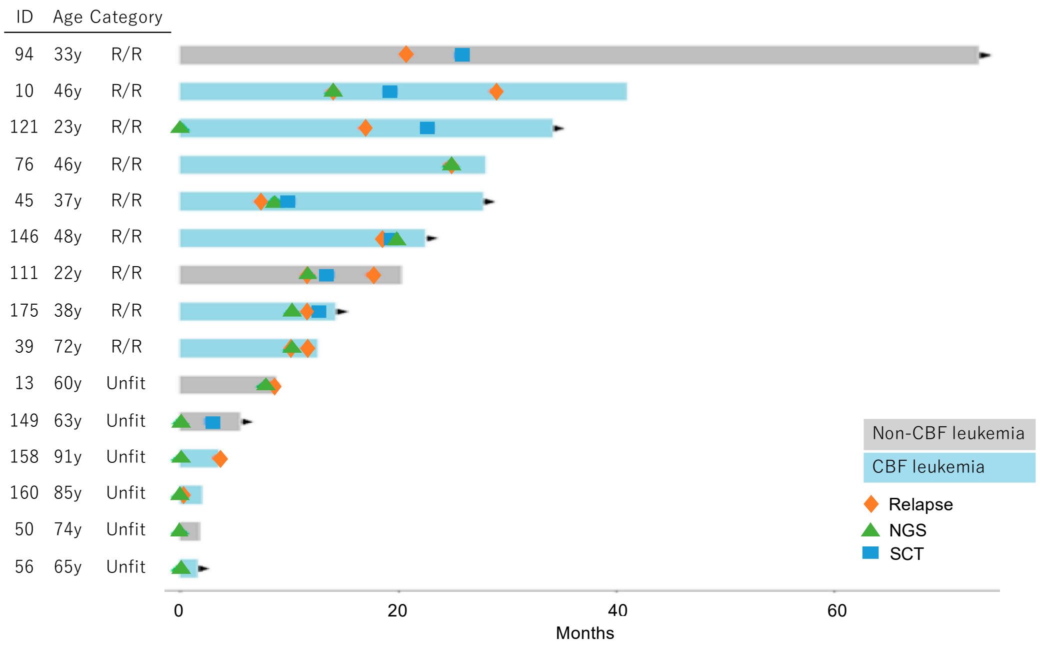

Supplemental Figure S2. Swimmer plot for 15 patients with a *KIT* mutation. Abbreviations: R/R: relapse/refractory, Unfit: unsuitable for the standard induction chemotherapy, CBF: core-binding factor**,** NGS: genome profiling by next-generation sequencing, SCT: stem cell transplant, y: years, ID: identification.
